# *Trichinella spiralis* Excretory–Secretory Products Stimulate Host Regulatory T Cell Differentiation through Activating Dendritic Cells

**DOI:** 10.3390/cells8111404

**Published:** 2019-11-07

**Authors:** Xi-Meng Sun, Kai Guo, Chun-Yue Hao, Bin Zhan, Jing-Jing Huang, Xinping Zhu

**Affiliations:** 1 Department of Medical Microbiology and Parasitology, School of Basic Medical Sciences, Capital Medical University, Beijing 100069, China; 2 Department of Pediatrics, National School of Tropical Medicine, Baylor College of Medicine, Houston, TX 77030, USA

**Keywords:** *Trichinella spiralis*, excretory–secretory products, CD4^+^ T cell, dendritic cells, regulatory T cells

## Abstract

*Trichinella spiralis* maintains chronic infections within its host, involving a variety of immunomodulatory properties, the mechanisms of which have not been completely elucidated. In this study, we found that *T. spiralis* infection induced strong regulatory T cell responses through parasite excretory–secretory (ES) products, characterized by increase of CD4^+^CD25^+^Foxp3^+^ and CD4^+^CD25^−^Foxp3^+^ Treg cells accompanied by high levels of IL-10 and TGF-β. *T. spiralis* adult worm excretory–secretory products (AES) and muscle larvae excretory–secretory products (MES) were both able to activate BMDCs in vitro to facilitate their maturation and to create regulatory cytokines IL-10 and TGF-β. The *T. spiralis* AES- and MES-pulsed dendritic cells (DCs) possessed abilities not only to present antigens to sensitized CD4^+^ T cell to stimulate their proliferation but also to induce naive CD4^+^ T cells to differentiate to Treg cells secreting IL-10 and TGF-β. The passive transfer of *T. spiralis* AES- and MES-pulsed bone marrow-derived dendritic cells (BMDCs) conferred the naive mice to acquire the differentiation of Treg cells. *T. spiralis* AES possesses a better ability to induce Treg cells than did MES, although the latter has the ability to induce CD4^+^CD25^−^Foxp3^+^ Treg cells. The results obtained in this study suggested that *T. spiralis* ES products stimulate the differentiation of host Treg cells possibly through activating dendritic cells to create a regulatory environment that benefits the survival of the parasite in the host.

## 1. Introduction

Parasitic helminths modulate host immune response for long-term survival and continued infection. Through the coordination of complex signaling networks that intimately link the host innate and adaptive immune systems, the host may achieve an efficient immune defense against invading pathogenic microorganisms. Upon interaction with certain antigens presented by dendritic cells (DCs), CD4^+^ T cells activate, expand, and differentiate into different effector subsets termed Th1, Th2, and Th17 cells and induced regulatory T cells (iTreg). The lymphocyte differentiation is predominantly governed by the cytokines in the microenvironment and, to some extent, by the strength of the interaction of the T cell receptor with the antigen [1]. For effective control of infections and avoidance of autoimmune and immunopathological diseases, the rigorous regulation of effector T cell responses is required. As a survival strategy of invading helminthic parasites, chronic helminths infection is often associated with polarized Th2 responses and stimulates Treg to produce regulatory and anti-inflammatory cytokines that reduce host immune responses [2]. The iTregs have essential roles in maintaining immune homeostasis by regulating these effector T cell responses, by facilitating pathogens immune evasion [3], and by preventing potentially pathogenic effects on the host. These effects are achieved through a variety of mechanisms [4,5,6].

Treg cells have suppressive activity on immune responses, but it had been documented that regulatory T cell populations remain diverse [7]. Treg cells expressing CD25 and Foxp3 are naturally present in the immune system and considered as negative regulators of immune response. These natural Treg cells originate during thymic development and appear first in fetal circulation [8]. Other cells such as IL-10-secreted Tr1 cells and TGF-β-secreted certain CD4^+^CD25^−^Foxp3^+^ T cells also have been found to possess regulatory activities [9]. Treg cells with Foxp3 marker are able to suppress the activation, proliferation, and effector functions (such as cytokine production) of natural killer (NK) cells and natural killer T (NKT) cells, B cells, CD4^+^ and CD8^+^ T cells, and antigen-presenting cells (APCs), etc. in vitro and in vivo [10]. Different helminth infections, such as *Heligmosomoides polygyrus* [11], *Schistosoma japonicum* [12], *Schistosoma mansoni* [13], and *Brugia malayi* [14] are known to provoke an increased number of Foxp3^+^ Tregs.

*Trichinella* is a fascinating infection model for induction and regulation of differentiation of various CD4^+^ T cells subsets for adaptive immune systems in response to infection. With the infection of *T. spiralis*, the entire life cycle is completed in the same host where the adult worm, newborn larva (NBL), and muscle larva (ML) are sequentially developed. NBL are released from sexually mature adult worms in the intestine, followed by the migration of NBL to skeletal muscle to develop capsulated infective ML [15]. Each of these life phases may uniquely affect host immune response but have not been adequately investigated to date. Indeed, even in the muscle phase of *T. spiralis* infection, larvae are “hidden” inside the cysts, which are made of transformed muscle cells called nurse cells surrounded by a collagen capsule [16] and communicated with the host through ML excretory/secretory (ES) products.

As other parasitic helminths, *T. spiralis* develops a sophisticated strategy to modulate host immune systems to avoid immune attack during the process of the parasite life stage transformation. Such a strategy must be carefully balanced in order to avoid compromising host survival [17]. However, the mechanisms underlying the immunomodulation induced by *T. spiralis* infection has not been completely elucidated. Although we have identified several molecules secreted by *T. spiralis* playing important roles in the modulation of host immune systems in our previous studies, there is few in vivo data available to support such a cross-regulation among Treg, Th1, Th2, and Th17 responses elicited by *T. spiralis* infection. These identified molecules include *T. spiralis* paramyosin (*Ts*Pmy) [18,19,20] and calreticulin [21] expressed on the surface of different stages of *T. spiralis* as well as in the ES products of adult and muscle larval worms with the ability to bind to their host complement components to avoid host complement attack. It has been also found that *Ts*Pmy modulates Treg differentiation to reduce host adaptive immune response as a *T. spiralis* survival strategy to live in its host [22]. Moreover, *T. spiralis* adult worm excretory–secretory products (AES) demonstrated a therapeutic potential for alleviating dextran sulfate sodium (DSS)-induced inflammatory colitis in mice with upregulation of Tregs and anti-inflammatory cytokines [23]. However, the mechanism underlying the upregulation of Treg response induced by *T. spiralis*-secreted products is not clear. In this study, we like to determine whether *T. spiralis* excretory–secretory products, especially AES, induce the differentiation of regulatory T cells through activating dendritic cells.

## 2. Materials and Methods

### 2.1. Animals

Specific pathogen-free (SPF) 6–8 week-old female BALB/c mice (*n* = 100 in total), 6–8 week old female ICR (Institute of Cancer Research) mice (*n* = 3 in total), and female Wistar (*n* = 10 in total) mice with weights of 150–200 g each were purchased from the Laboratory Animal Services Center of the Capital Medical University (Beijing, China). All animal procedures were approved by the Capital Medical University Institutional Animal Care and Use Committee (IACUC; Permit Numbers: AEEI-2015-183 and AEEI-2015-184). All mice were maintained under SPF conditions with humidity of 70 ± 10% and temperature of 20 ± 2 °C, and animal protection and care principles were performed in all animals experiments according to the National Institutes of Health (NIH) and IACUC guidelines for the care and use of laboratory animals.

### 2.2. Parasites and Antigens Preparation

*T. spiralis* (ISS 533 strain) was cultured in female ICR mice, and muscle larvae (ML) were isolated from the muscles of infected ICR mice by a previously described method of modified pepsin–hydrochloric acid digestion [24]. Adult *T. spiralis* worms were obtained from the intestines of the Wistar mice at 106 h (4.5 days) after each being orally infected with 12,000 muscle larvae [25]. The excretory–secretory products of *T. spiralis* ML (MES) and adult worms (AES) were prepared and collected as previously described [23,26,27]. *T. spiralis* ML were freshly collected from mice muscle on day 40 post-infection and washed three times with phosphate-buffered saline (PBS) and then cultured in Roswell Park Memorial Institute (RPMI)-1640 medium (Hyclone, Logan, UT, USA) supplemented with 100 U/mL penicillin and 100 U/mL streptomycin (Solarbio, Beijing, China) at 37 °C, 5% CO_2_ for 48 h. The culture supernatants containing MES products were concentrated by centrifugation and buffer exchanged into PBS and then filtered through a 0.45-micron syringe filter (Merck, Darmstadt, Germany). AES was obtained by culturing adult worms in medium as described for MES collection. The protein concentrations of the prepared *T. spiralis* ES antigens were determined by bicinchoninic acid (BCA) assay (Merck). The contaminated endotoxin was removed by ToxOut™ High Capacity Endotoxin Removal Kit (Biovision, San Francisco, CA, USA). All antigens were stored at −80 °C. Lipopolysaccharide (LPS) (Sigma-Aldrich, St. Louis, MO, USA) was used as a positive control for immune response in vitro.

### 2.3. Experimental Infection

To analyze T cell differentiation and the related-cytokine responses during different stages of *T. spiralis* infection, each BALB/c mouse was orally infected with 400 infective *T. spiralis* ML. On day 6, 15, 30, and 60 post-infection, four mice were randomly selected from the infected group and control group and were sacrificed. Spleens were collected, and splenocytes were harvested. Day of collecting was chosen according to the developing stage of the worm in the host. Day 6 was the intestinal phase of *T. spiralis*; day 15 was newborn larvae migrating in the circulatory system and tissue of host; day 30 was the larva capsule formation phase; and day 60 was the convalescent phase.

### 2.4. Cell Culture

Single cell suspensions of splenocytes were prepared in mouse lymphocyte separation medium according to the manufacturer’s instructions (Dakewe Biotech, Shenzhen, China). Subsequently, 2 × 10^5^/well splenocytes in 200 μL of RPMI-1640 media containing 10% fetal bovine serum (FBS; Thermo Fisher, Life Technologies, Carlsbad, CA, USA) and 100 U of penicillin/streptomycin/mL were cultured in 96-well plates at 37 °C, 5% CO_2_ for 72 h in the presence of 25 ng/mL phorbol 12-myristate 13-acetate (PMA; Sigma-Aldrich) and 1 mg/mL ionomycin (Sigma-Aldrich). Culture supernatants were recovered for detecting different cytokines using corresponding ELISA kits for detecting IL-10, TGF-β, or IL-21 using the eBioscience ELISA Ready-SET-Go kit (eBioscience, San Diego, CA, USA) and for detecting other cytokines using Mouse ELISA KIT (Dakewe Biotech) according to the manufacturer’s instructions.

### 2.5. Generation of Bone Marrow-Derived Dendritic Cells (BMDCs)

Mouse BMDCs were generated from 6 to 8 week-old BALB/c mice according to the procedure described by Ilic et al. [26] with slight modifications. Bone marrow cells were collected from the femur bones of BALB/c mice and passed through a 100-μm Falcon cell strainer (Solarbio) to remove debris and clumps. Red blood cells were lysed using ACK (Ammonium-Chloride-Potassium) lysis buffer (Tiangen, Beijing, China). The marrow cell fraction was washed with complete RPMI 1640 medium supplemented with 10% FBS and penicillin/streptomycin. Total of 2 × 10^6^ cells was resuspended in 2 mL complete RPMI 1640 medium in each well of 6-well plates and cultured at 37 °C, 5% CO_2_ for 3 h. After removing the nonadherent cells, the remaining adherent cells were cultured in RPMI 1640 medium, which contains growth factors of 10 ng/mL recombinant granulocyte-macrophage colony-stimulating factor (GM-CSF) (Peprotech, Rocky Hill, NJ, USA), 2 ng/mL IL-4 (Peprotech), and 10% FBS for 6 days with fresh medium change on day 3 and day 5. The immature BMDCs were harvested for further experiment on day 6.

### 2.6. In Vitro BMDCs Activation Assay

The harvested immature BMDCs generated above were cultivated in 6-well plates in complete RPMI-1640 medium with stimulation of AES (10 µg/mL) or MES (10 µg/mL) at 37 °C, 5% CO_2_ for 48–72 h. The same cell culture with 2 µg/mL LPS was used as a positive control and with bovine serum albumin (BSA) (10 µg/mL) (Thermo Fisher, Life Technologies) or PBS as a negative control. The stimulated BMDCs were washed and resuspended in 200 µL PBS containing 2 µL rat anti-mouse CD16/CD32 antibody (BD PharMingen, San Jose, CA, USA) for Fc receptors (FcR) block and then stained with P-phycoerythrin (PE)-labeled anti-mouse CD11c antibodies (BD PharMingen) and fluorescein isothiocyanate (FITC) anti-mouse CD80, CD86, CD40, or MHCII (BD PharMingen) for fluorescence-activated cell sorting (FACS) analysis. The cytokine levels in the BMDCs culture supernatants were determined by corresponding ELISA kits.

### 2.7. Co-Incubation of CD4^+^ T cells with T. spiralis ES-Primed BMDCs

For evaluation of T cells proliferation and polarization by *T. spiralis* ES (*Ts*ES)-primed BMDCs, the *T. spiralis*-sensitized CD4^+^ T cells were collected from the spleens of BALB/c mice infected with *T. spiralis* using magnetic-activated cell sorting (MACS) with mouse CD4^+^ T cells isolation kits following manufacturer’s instructions (Miltenyi Biotec, Bergisch Gladbach, Germany), and naive CD4^+^ T cells were isolated from the spleens of naive BALB/c mice by the same protocol as described above. A total of 5 × 10^5^ *T. spiralis*-sensitized or naive CD4^+^ T cells were co-cultivated with 2.5 × 10^4^ *Ts*ES-primed BMDCs in each well of a 96-well plate and labeled with 5- and 6-carboxyfluorescein diacetate succinimidyl ester (CFSE) (eBioscience). In the *T. spiralis*-sensitized and naive T cell culture, 5 µg/mL anti-CD3 (Peprotech) and 5 µg/mL anti-CD28 (Peprotech) were added as signal one and a costimulatory signal two without leading to early cell death for proliferated cells. For BMDCs/*T. spiralis*-sensitized T cells co-cultures for 72 h at 37 °C, 5% CO_2_, the proliferation of T cells was measured by fluorescence-activated cell sorting (FACS). The co-incubated BMDCs/naive T cells were continued for 40 h, and cells were recovered for detecting the percentage of CD4^+^CD25^+^Foxp3^+^ T cells in total CD4^+^ T cells. In addition, in order to determine the cytokine production, the co-incubation supernatants were recovered for measuring cytokine release by ELISA as described above.

### 2.8. Adoptive Transfer of TsEs-primed DCs into Naive Mice

After being incubated with AES or MES (10 µg/mL) in vitro for 48 h, the mouse BMDCs were washed twice in RPMI 1640 and then resuspended in PBS. Each naive BALB/c mouse in a group of 30 mice was injected intravenously with 2 × 10^6^ DCs. The DCs incubated with BSA and PBS were used as nonrelevant protein or negative controls, respectively. One week after the transfer, splenocytes from all the mice were harvested for the analysis of the presence of Tregs and the secretions of the Th1 (IFN-γ), Th2 (IL-4), and Th17 (IL-17) cytokines.

### 2.9. TsES-Induced T cells Responses in Mice

BALB/c mice were divided randomly into 3 groups with 4 mice in each group, and each group was immunized intraperitoneally with 200 µg AES, 200 µg MES, or 200 µL PBS twice with 14-day interval. Two weeks after the last immunization, splenocytes from all the mice were harvested for the analysis of cytokine production and the presence of Tregs, Th1, Th2, and Th17 cells. Meanwhile, the splenocytes from AES-immunized mice were surface stained with anti-mouse CD3-PerCP-eFluor^®^ 710 (eBioscience) and anti-mouse CD4-FITC (eBioscience). Intracellular staining was done using anti-mouse Foxp3-PE. The CD4^+^CD25^+^ T cells, CD4^+^CD25^−^ T cells, and Foxp3^+^ T cells were sorted using the MoFlo XDP cell sorter (Beckman Coulter, Inc, Fullerton, CA, USA).

The CD4^+^CD25^+^ T cells, CD4^+^CD25^−^ T cells, and CD4^+^Foxp3^+^ T cell samples were stored in Trizol reagent (Life Technologies, Rockville, MD, USA) to extract total RNA. The Treg-related transcription factors and cytokines gene expression were evaluated via real-time PCR. 

### 2.10. Intracellular Cytokines and Foxp3 Staining

For FACS analysis of Th1, Th2, or Th17 cells, the harvested splenocytes were stimulated with 25 ng/mL PMA, 1 µg/mL ionomycin, and 0.66 µL/mL Golgistop^TM^ (BD PharMingen) in complete RPMI 1640 medium for 6 h in 6-well plates at 37 °C, 5% CO_2_, and then, the harvested cells were FcR blocked with rat anti-mouse CD16/CD32 antibody and stained as follows: surface stained with anti-mouse CD3-APC-eFluor^®^ 780 (eBioscience) and anti-mouse CD4-FITC (eBioscience) and intracellularly stained with anti-mouse IFN-γ-PE-Cyanine7, anti-mouse IL-4-PE-Cyanine7, or anti-mouse IL-17A-PE-Cyanine7 (or isotype IgG2a, IgG1 control antibody) (eBioscience). Cytofix/Cytoperm buffer was used in the intracellularly staining process (BD PharMingen) for washing, fixing, and permeabilizing cells.

For determination of Tregs, the harvested splenocytes from each mouse were directly FcR antibody blocked and stained with mouse regulatory T cells staining kit according to manufacturer’s instructions (eBioscience). The kit contained stains for antibodies: anti-mouse CD4-FITC, anti-mouse CD25-APC, and anti-mouse Foxp3-PE. The surface marker staining used was PerCp-labeled anti-CD3, FITC-labeled anti-CD4, and APC-labeled anti-CD25. After cells were washed, fixed, and permeabilized, intracellular staining was performed using PE-labeled anti-Foxp3 antibody or IgG2a rat immunoglobulin control antibody. Data were analyzed with Flow software (BD Biosciences, San Jose, CA, USA).

### 2.11. Quantitative Real-Time PCR

The relative changes in expression of Foxp3, TGF-β1, and IL-10 genes in CD4^+^CD25^+^, CD4^+^CD25^−^, and CD4^+^Foxp3^+^ T cells were evaluated via real-time quantitative PCR. Table 1 shows the primer sequences for each detected gene. Total RNA was extracted from T cells by Trizol reagent method in accordance with the manufacturer’s protocols (Life Technologies). After synthesizing cDNA from total RNA using the PrimeScript 1^st^ Strand cDNA Synthesis Kit (Takara, Kusatsu, Japan), real-time quantitative PCR reactions were performed using TransStart Top/Tip Green qPCR SuperMix (TransGen, Beijing, China). The mRNA expression in each sample was finally determined after correction with Hypoxanthine-guanine phosphoribosyltransferase (HPRT) 1 expression. Results were calculated using the 2^−∆∆Ct^ method [28]. In brief, the gene-specific threshold cycle (Ct) for each sample (∆Ct) was corrected by subtracting the ∆Ct for the HPRT 1 housekeeping gene. PBS controls were selected as the reference samples, and the ∆Ct for all experimental samples was subtracted from the ∆Ct for the control samples (∆Ct):

∆∆Ct = (CT_Target_ − CT_Hprt 1_)_Time x_ − (CT_Target_ − CT_Hprt 1_)_Time 0_ (1)

where Time x is any time point and Time 0 represents the 1× expression of the target gene normalized to HPRT 1.

### 2.12. Statistical Analysis

All data were presented as means ± SDs (standard deviations). Statistical significance was calculated with two-tailed *t*-test, one-way analysis of variance (ANOVA), or student’s unpaired using the GraphPad Prism version 6 software (San Diego, CA, USA), and *p* < 0.05 was considered significant.

## 3. Results

### 3.1. T. spiralis Infection Induced Differentiation of Th2 and Treg Cells

**Figure 1:**
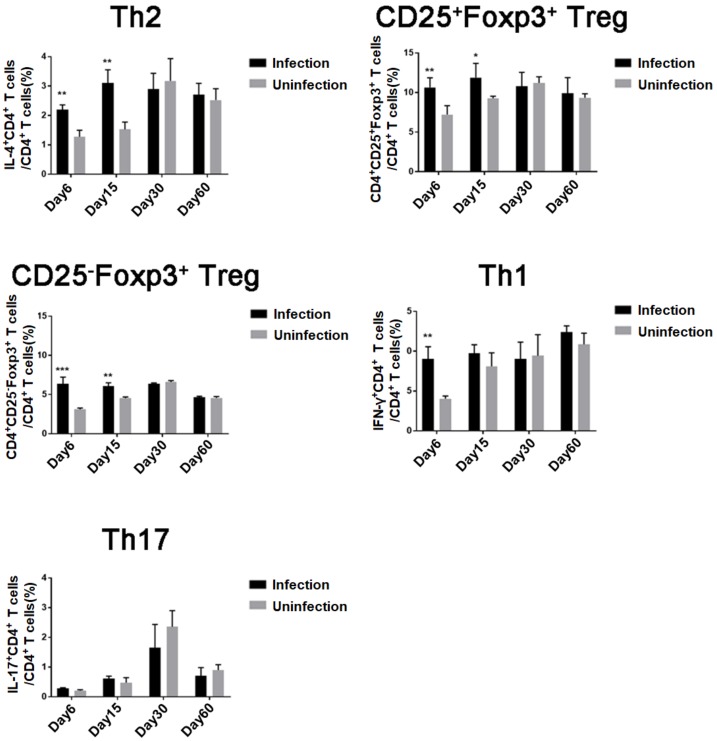
Differentiation of Treg, Th1, Th2, and Th17 cells in mice during *T. spiralis* infection: For each of three independent experiments, 16 female BALB/c mice were each infected with 400 *T. spiralis* ML. Four mice were randomly sacrificed at different stages post-infection (intestinal phase, day 6; newborn larva (NBL) migration, day 15; larva capsule, day 30; and convalescent phase, day 60); Th2, Treg, Th1, and Th17 CD4^+^ T cells in the spleen were measured by fluorescence-activated cell sorting (FACS). Data are shown as means ± SDs; * *p* < 0.05; ** *p* < 0.01; *** *p* < 0.001.

To analyze the effect of *T. spiralis* infection on CD4^+^ T cells differentiation, the subpopulations of CD4^+^ T cells isolated from mouse spleens were measured by FACS during different infection stages (Figure 1). The Th2 CD4^+^ T cells expressing IL-4 and Treg cells expressing CD4^+^CD25^+^Foxp3^+^ and CD4^+^CD25^–^Foxp3^+^ were significantly induced at the early intestinal stage (6 days post-infection, day 6, Figure S1) and NBL migration stage (day 15, Figure S1) compared to the those of uninfected mice. The increase of Th1 CD4^+^ cells expressing IFN-γ was only observed at the early intestinal infection stage (day 6). After adult worms were expelled from intestine and the NBL migrated to muscle to establish encapsulated muscle larvae (days 30–60, Figure S1), all Th1, Th2, and Treg cells were recovered to normal level (no significant difference with uninfected mice). Concurrently, IL-17A^+^ Th17 cells were not significantly increased during infection. These results indicated that *T. spiralis* infection mainly induced Th2 and Treg responses during the early infection of *T. spiralis* and that the maintenance of Th2 and Treg cells must be consistently stimulated by worm infection. After adult worms were expelled from the intestine and muscle larvae were encapsulated, no host cellular immune response was observed.

### 3.2. T. spiralis Infection Increased the Expression of Inhibitory Cytokines

**Figure 2:**
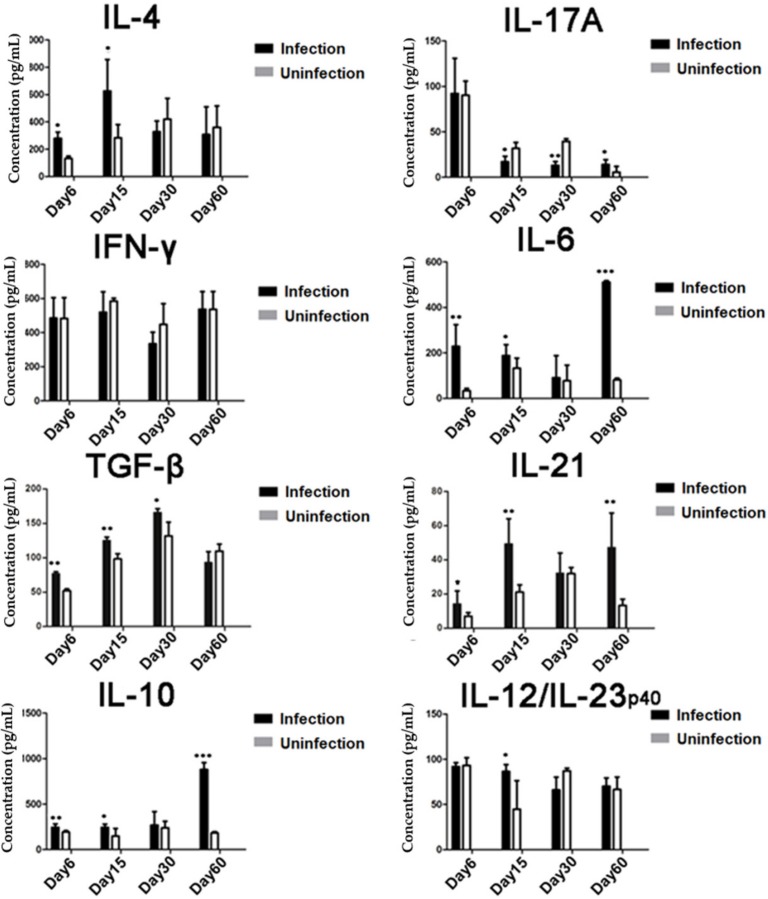
Cytokine profile measured in the culture of splenocytes isolated from *T. spiralis*-infected mice: On days 6, 15, 30, and 60 after infection with *T. spiralis*, four infected mice at each time point were sacrificed and splenocytes were isolated. The culture supernatants were collected after being incubated with phorbol 12-myristate 13-acetate (PMA) and ionomycin for 72 h, and the concentrations of IL-4, IL-17A, IFN-γ, IL-6, TGF-β, IL-21, IL-10, and IL-12/23p_40_ were measured by ELISA. Data are representative of three independent experiments, expressed as means ± SDs, * *p* < 0.05; ** *p* < 0.01; *** *p* < 0.001, compared to the uninfected group.

Similar to the cellular differentiation, the cytokine profiles secreted by the splenocytes reflected the induction of Th2 and Treg responses in infected mice. The levels of IL-4, IL-10, and TGF-β were significantly increased in the culture of splenocytes isolated from mice infected with *T. spiralis* during the early stages (days 15–30) compared to the level secreted by the splenocytes of uninfected normal mice. The level of IL-10 remained high during the entire infection period and reached its highest level on day 60. Interestingly, the levels of IL-6, IL-12, and IL-21 were also increased during the early stages of infection and IL-6 and IL-21 reached their highest levels on day 60. There were no significant increases in the levels of IFN-γ and IL-17 during infection. These results indicate that *T. spiralis* infection stimulated regulatory immune responses resulting in increased Treg cells and regulatory cytokines TGF-β, IL-10, and IL-21 (Figure 2)**.**

### 3.3. Immunization with AES or MES Induced Treg Differentiation in Mouse

**Figure 3:**
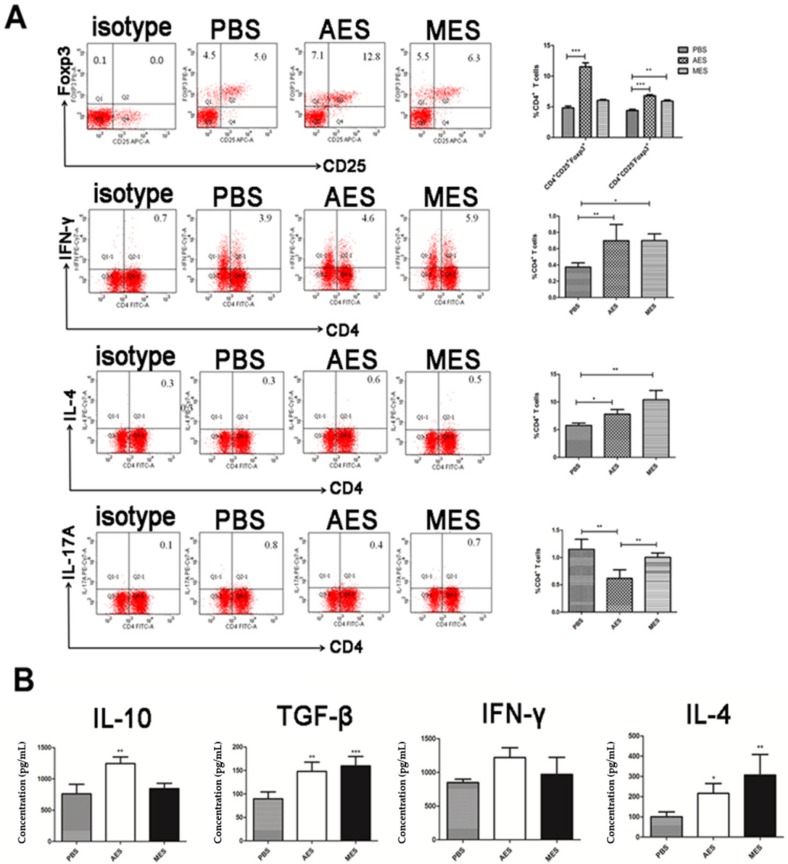
The differentiation of Th1, Th2, Th17, and Treg cells and secreted cytokines in mice immunized with adult worm excretory–secretory products (AES) or muscle larvae excretory–secretory products (MES): (**A**) The splenocytes were isolated from mice immunized with AES and MES sorted for CD4, CD25, Foxp3 and Th1, Th2, and Th17 by FACS. (**B**) Splenocyte supernatants were harvested after being cultured 72 h with PMA/ionomycin, and the cytokine profile in the culture was examined by ELISA. Results are expressed as means ± SDs from 3 independent experiments. * *p* < 0.05; ** *p* < 0.01; *** *p* < 0.001, compared to phosphate-buffered saline (PBS) or indicated control.

To evaluate the role of *T. spiralis* ES products on Treg cell differentiation in vivo, BALB/c mice were immunized intraperitoneally with AES or MES and the CD4^+^ T cells isolated from splenocytes of immunized mice were measured by FACS. Similar to *T. spiralis* infection, AES immunization significantly increased the percentage of CD4^+^CD25^+^Foxp3^+^ and CD4^+^CD25^−^Foxp3^+^ Treg cells; however, MES only significantly increased CD4^+^CD25^−^Foxp3^+^ Treg cells. Both AES and MES upregulated CD4^+^ T cells expressed IFN-γ^+^ (Th1) and IL-4^+^ (Th2) but not IL-17A^+^ (Th17) (Figure 3A). Also as shown in Figure 3A, the cytokine profiles secreted by splenocytes of mice immunized with AES showed high levels of regulatory cytokine IL-10 and TGF-β, consistent with the increased population of CD4^+^CD25^+^Foxp3^+^ Treg cells. The cytokines IFN-γ and IL-4 were also highly observed in the supernatant of cell culture of AES-immunized mice. MES immunization only stimulated IL-4 and TGF-β (Figure 3B). The findings above confirmed that, similar to the *T. spiralis* infection, *Ts*ES stimulated mixed Th1/Th2 responses along with promoting Treg differentiation, mainly by adult worm excretory–secretory products.

### 3.4. The mRNA Expression Profile Further Confirmed that AES Immunization Upregulated the Expressions of Foxp3, TGF-β, and IL-10

**Figure 4:**
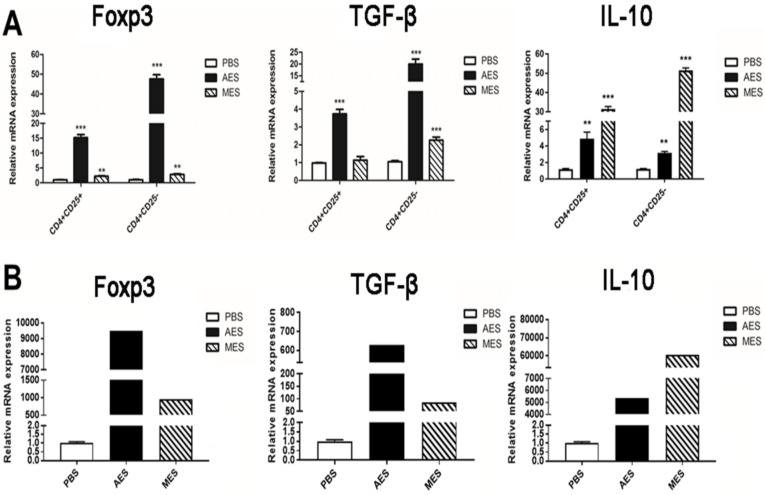
mRNA expression profile for Treg-related marker proteins in mice immunized with AES and MES: BALB/c mice were immunized with AES, MES, or PBS. (**A**) The CD4^+^CD25^+^ and CD4^+^CD25^−^ T cells were acquired by FACS, and the mRNA for Foxp3^+^, TGF-β, and IL-10 was measured by RT-PCR. (**B**) CD4^+^Foxp3^+^ T cells were isolated by FACS for mRNA analysis of Foxp3, IL-10, and TGF-β. Results are presented as means ± SDs from three independent experiments normalized to the level of HPRT 1. ** *p* < 0.01; *** *p* < 0.001, compared to the PBS control.

As CD4^+^Foxp3^+^ T cells have different CD25 expressions, effects of AES or MES on the mRNA expression of transcription factors and cytokines in CD4^+^CD25^+^ and CD4^+^CD25^−^ T cells from BALB/c mice spleens in vivo were examined. The expression level with Foxp3, a master transcription factor for the development and function of regulatory T cells, was upregulated for *T. spiralis* ES groups. Comparing the PBS group with the higher expression in the AES group, the evidence shows AES preferentially induces Foxp3^+^ Treg cells to likely produce higher levels of TGF-β and IL-10, which are the anti-inflammatory cytokines produced by Treg that play important roles in host defense. The mRNA expressions of TGF-β and IL-10 were elevated with *T. spiralis* ES immunization. AES mainly induced Foxp3+ and TGF-β, and MES mainly induced IL-10 (Figure 4). The results indicated that functional Tregs were induced better by AES than by MES (Figure 4A). Further experiments with AES confirmed that AES significantly induced the expressions of Foxp3^+^, IL-10, and TGF-β mRNA in CD4^+^Foxp3^+^ T cells (Figure 4B). Together, these findings support a conclusion that *Ts*ESs, especially AES, upregulate the differentiation of Treg cells and the secretion of regulatory cytokines IL-10 and TGF-β in both protein and mRNA expression levels.

### 3.5. T. spiralis ES Induces Maturation of BMDCs

**Figure 5:**
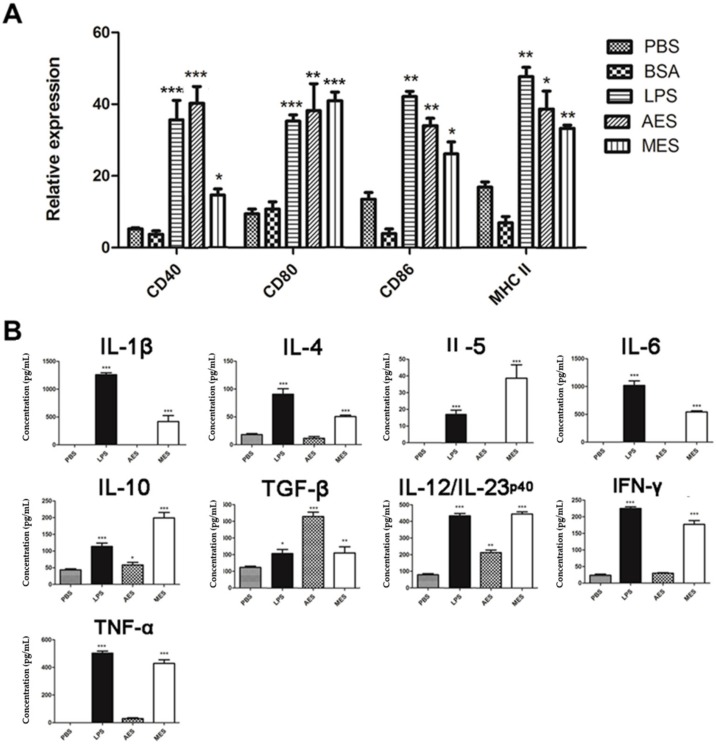
Maturation of Bone Marrow-Derived Dendritic Cells (BMDCs) pulsed by *T. spiralis* excretory/secretory (ES) products: Mouse BMDCs were cultured for 48 h with PBS, BSA, lipopolysaccharide (LPS), MES, or AES. (**A**) Surface markers (CD40, CD80, CD86, and MHC II) on DCs were sorted by FACS and the percentage of surface markers expression on CD11^+^ DCs stimulated with the indicated antigens. (**B**) Cytokines secreted by *T. spiralis* ES-stimulated BMDCs in the culture supernatant were measured by ELISA. Results are presented as means ± SDs from three individual experiments. * *p* < 0.05; ** *p* < 0.01; *** *p* < 0.001, compared to the PBS group.

To investigate whether *T. spiralis* ES induces the maturation of DCs, the mouse BMDCs were stimulated with *T. spiralis* ES antigens for 48 h and surface markers for DC maturation were examined by FACS. We found that AES and MES of *T. spiralis* significantly upregulated the expressions of CD40, CD80, CD86, and MHC II on CD11c^+^ DCs, as did the positive control LPS, when compared to PBS control (Figure 5A). As a nonrelevant protein control, BSA had no effect on the maturation of DCs. These data indicate that *T. spiralis*-secreted AES or MES were able to induce the maturation of mouse BMDCs. The cytokine profile secreted by treated BMDCs demonstrated that both AES and MES significantly stimulated the secretion of regulatory cytokines IL-10 and TGF-β and of cytokine IL-12/23p40. MES also stimulated some other Th1/Th2 cytokines such as IL-1β, IL-4, IL-5, IL-6, INF-γ, and TNF-α. (Figure 5B). These results indicated that stimulation with *T. spiralis* AES and MES activated DCs to create a microenvironment of regulatory factors (IL-10 and TGF-β) which may benefit Treg cell induction.

### 3.6. TsES-Pulsed BMDCs Presents Antigens to T Cells

**Figure 6:**
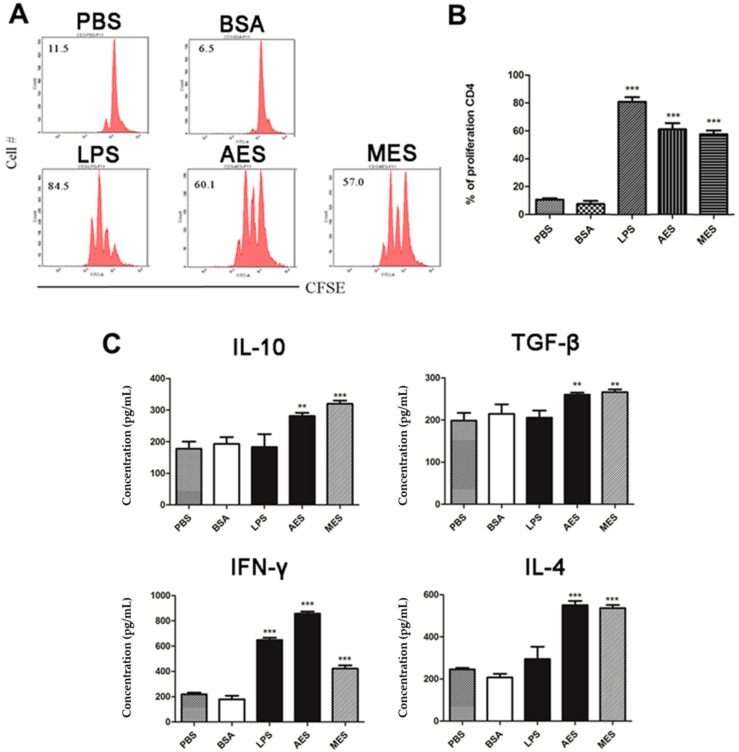
Proliferation and cytokines production of T cells in response to *T. spiralis* ES (*Ts*ES)-treated DCs: CD4^+^ T cells were isolated from splenocytes of *T. spiralis*-infected BALB/c mice, labeled with CFSE, and co-cultured for 72 h with BMDCs (stimulated with PBS, BSA, LPS, AES, or MES) in the presence of anti-CD3/anti-CD28. (**A**,**B**) FACS of T cells proliferation from one experiment of three independent experiments is presented. (**C**) Cytokine levels were measured in cell culture supernatants using ELISA. Data are expressed as means ± SDs from three independent experiments. Each experiment was done in triplicates. ** *p* < 0.01; *** *p* < 0.001, compared to the PBS control.

To assess whether *Ts*ES-stimulated BMDCs have the ability to present antigens to memory T cells, *T. spiralis*-sensitized CD4^+^ T cells were acquired from spleens of mice infected with *T. spiralis* and then co-cultured with AES- or MES-stimulated DCs for 72 h in the presence of anti-CD3/anti-CD28. Results demonstrated that *T. spiralis*-sensitized CD4^+^ T cells were highly proliferated when incubated with BMDCs primed with AES or MES but not with BMDCs incubated with PBS only. The results indicate that *Ts*ES-primed BMDCs enable to present *T. spiralis* antigens in AES and MES to memory CD4^+^ T cells that have been sensitized by *T. spiralis* infection (Figure 6A,B)**.** Concurrently, the memory response of *T. spiralis*-sensitized CD4^+^ T cells to the antigen presented from primed BMDCs produced high levels of IL-4, IFN-γ, TGF-β, and IL-10 (Figure 6C), indicating that the antigen presentation induced mixed Th1/Th2/Treg responses in sensitized CD4^+^ T cells. LPS-stimulated BMDCs only induced IFN-γ production. The results of proliferation and cytokine profiling clearly showed that *T. spiralis*-activated DCs enabled to present antigens to sensitized T cells to induce memory activation, with mixed Th1/Th2/Treg responses.

### 3.7. TsES-Primed DCs Induced Treg Cells In Vitro

**Figure 7:**
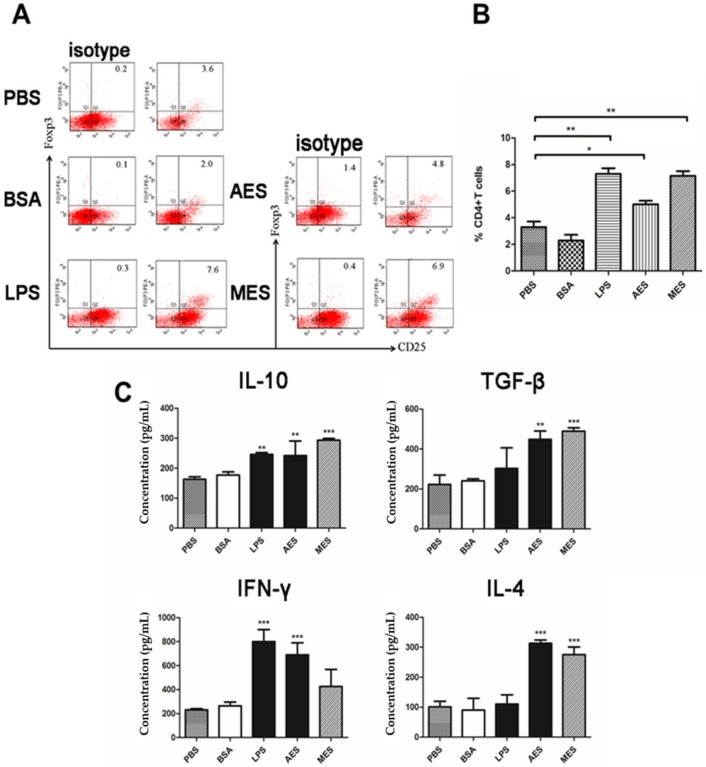
*Ts*ES-primed BMDCs upregulated Foxp3^+^ CD4^+^CD25^+^ T cells populations and Treg cytokines in naive T cells. T cells were isolated from naive BALB/c mice splenocytes and co-cultured with AES-, MES-, LPS-, BSA-, or PBS-treated BMDCs in the presence of anti-CD3/anti-CD28 for 48 h. (**A**) The cells were sorted with CD3, CD4, CD25, and Foxp3 by FACS. (**B**) Percentage of CD25^+^Foxp3^+^ cells in a CD4^+^ T cell population is shown. (**C**) Cytokines in co-cultured supernatants were detected by ELISA. Data are shown as the means ± SDs for three independent experiments. Results represent one of three independent experiments. * *p* < 0.05; ** *p* < 0.01; *** *p* < 0.001, compared to PBS or BSA control.

In order to evaluate whether *T. spiralis* ES antigens could induce Treg cells in vitro, the population of CD4^+^CD25^+^Foxp3^+^ T cells was measured in CD4+ T cells of naive BALB/c mice after being cultivated with AES- or MES-primed BMDCs. We found that AES- and MES-primed BMDCs significantly increased the percentage of CD4^+^CD25^+^Foxp3^+^ T cells compared to the BMDCs incubated with PBS or BSA only (Figure 7A,B). LPS also elevated the proportion of CD4^+^CD25^+^Foxp3^+^ T cells, possibly associated with LPS-induced tolerogenic BMDCs. Cytokines profiles of co-cultured supernatants also demonstrated increased IL-10 and TGF-β of regulatory cytokines, as well as IL-4, IFN-γ, and IL-17A (Figure 7C).

### 3.8. TsAES-Pulsed DCs Upregulated Treg Cells In Vivo

**Figure 8:**
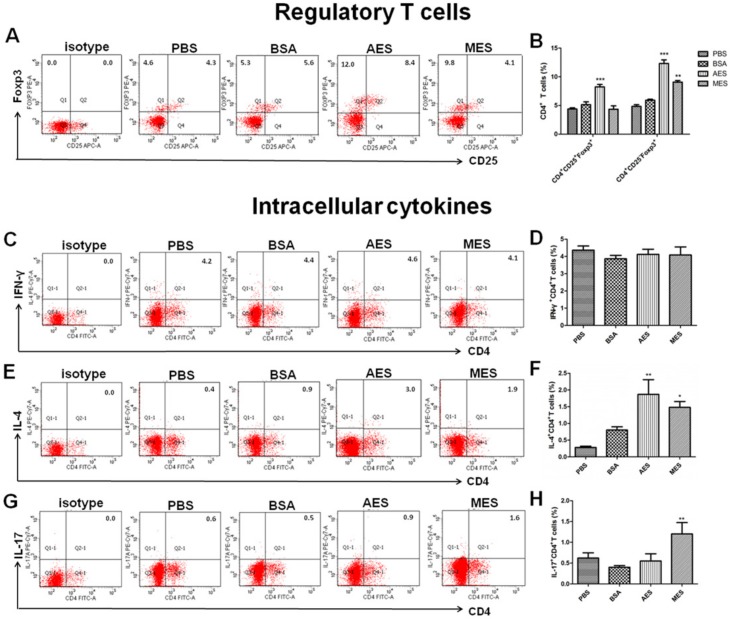
*Ts*ES-pulsed DCs induced Treg cell differentiation and Th2 cytokine in naive CD4^+^ T cells in vivo. The splenocytes were isolated from naive BABL/c mice passively transferred with *Ts*AES-, *Ts*MES-, PBS-, and BSA-treated BMDCs. (**A**) The frequencies of CD4^+^CD25^+^Foxp3^+^ and CD4^+^CD25^-^Foxp3^+^ T cells were measured by gated flow cytometry. (**B**) The percentages of CD4^+^CD25^+^Foxp3^+^ T cells and CD4^+^CD25^-^Foxp3^+^ T cells are presented as the mean ± SEM of five individual mice per group. (**C**,**D**) Frequencies of CD4^+^ T cells producing IFN-γ^+^, (**E**,**F**) frequencies of CD4^+^ T cells producing IL-4^+,^ and (**G**,**H**) frequencies of CD4^+^ T cells producing IL-17A^+^ are shown. The data are presented as the mean ± SEM of three independent experiments. * *p* < 0.05; ** *p* < 0.01; *** *p* < 0.001 in a one-way analysis of variance followed by Bonferroni correction.

To determine whether *Ts*ES-pulsed DCs could drive the differentiation of CD4^+^CD25^+^Foxp3^+^ T cells in vivo, *Ts*ES-primed DCs were passively transferred into naive mice. The levels of CD4^+^CD25^+^Foxp3^+^ Tregs in the spleen of those mice passively transferred with *Ts*AES-pulsed DCs were significantly elevated compared to those that received PBS or BSA-pulsed BMDCs, while CD4^+^CD25^+^Foxp3^+^ Treg populations did not change in mice who received *Ts*MES-pulsed DCs (Figure 8A,B), even though *Ts*MES-pulsed DCs induced significantly higher CD4^+^CD25^-^Foxp3^+^ Treg (*p* < 0.01) compared to the PBS control. Passive transfer of both *Ts*MES- and *Ts*AES-pulsed BMDCs induced high levels of IL-4 and IL-17A but not INF-γ, compared to the level of mice that received PBS-treated BMDCs (Figure 8C–F). The results suggest that helminth excretory–secretory products upregulate CD4^+^CD25^+^Foxp3^+^ Treg and Th2 polarization possibly through programming DCs.

## 4. Discussion

In this study, we have identified that *T. spiralis* infection induced the generation of both CD4^+^CD25^+^Foxp3^+^ and CD4^+^CD25^−^Foxp3^+^ Treg cells in the early infection stages including the intestinal phase and the NBL migration phase, associated with high levels of regulatory cytokines IL-10, TGF-β, and IL-21. The results are consistent with other helminth infections that stimulated Foxp3^+^ Treg cells. Long-term persistence of *H. polygyrus* infection was associated with enhanced frequencies of Treg cells and associated with IL-10 and TGF-β production [11]. Both *B. malayi* larvae and adults induced Treg cells accompanied by raised CTLA-4, CD25, and CD103 expression [14]. Likewise, filarial helminth *Litomosoides sigmodontis* infection induced hyporesponsiveness that can be reversed by depletion of Treg cells in mice [29]. Treg cells and regulatory cytokines IL-10 and TGF-β participated in immune evasion of helminth infection by negative regulation of the host immune system.

In addition, *T. spiralis* infection is characterized by induction of Th1 and Th2 response in the intestinal phase in which *T. spiralis* rapidly develops to adult worms. The mature adult worms produce newborn larvae, which disseminate through blood circulation to the muscles. The larvae migration stimulates strong Th2 response that results in the expulsion of adult worms and NBL from the intestine [2,30]. In this study, we confirmed that the intestinal phase of *T. spiralis* induced mixed Th1/Th2 responses as reflected by the increased CD4^+^ T cells that expressed IFN-γ (Th1) and IL-4 (Th2) as well as increased levels of IFN-γ and IL-4. When the larvae migrated to muscle, we observed the predominant Th2 response with high levels of IL-4- and IL-4-expressed CD4^+^ cells. After larvae moved to muscle and ML, all Th1/Th2/Treg responses returned to normal, possibly due to encapsulation and isolation of ML from host immune systems. In our study, we observed the slight increase of Th17 response at the early infections of the intestinal adult stage and the larval migration stage, and it is correlated with Th2 responses and worm expulsion. The results are consistent with the observation that DC-activated TGF-β induced Th17 to stimulate the intestinal contractility and the expulsion of *T. spiralis* worms [31], and then, Th17 returned to normal or even decreased at the late stage of infection. It is possible that early *T. spiralis* infection induces the Th2 and Th17 responses that result in the expulsion of adult worms from intestines. The increased TGF-β may contribute to the early increase of Th17 and may strongly contribute to the stimulation of Treg that inhibits inflammation and benefits worm’s survival. The reciprocal development of Th17 and Treg responses during infection is important for the balance of immune protection and immunopathology [32]. TGF-β was also involved in the control of local inflammation in infected muscle and promotes parasite survival [33]. The results showed that *T. spiralis* infection induced the differentiation of Treg cells that may play an immunomodulatory role and inhibits inflammatory response for parasite persistence in hosts during *T. spiralis* infection.

As multicellular pathogens, helminth induce and regulate host immune responses through their exposed antigens recognized by the host immune system. Parasitic helminth ES proteins are the major components of antigens that stimulate host immune responses [26]. ES products from different stages of *T. spiralis* regulated host immune responses at the macrophage level in vitro by inhibiting pro-inflammatory cytokine production, by boosting the expression of anti-inflammatory cytokines IL-10 and TGF-β, and by inducing macrophages toward the alternative phenotype, all of which may play important roles in the worm survival in the host and protect hosts from unnecessary damage caused by excessive inflammatory responses [34].

DCs are highly specialized antigen-presenting cells that are important not only in initiating immune responses but also in regulating the magnitude and differentiation of the immune responses [35]. For infection, pathogens and/or their products may interact with DCs through various families of pattern recognition receptors, such as Toll-like receptors [36,37], facilitating DC maturation which is the basis for the generation and polarization of the adaptive immune responses. Different helminth-derived molecules from one particular helminth or from different developmental stages of worms, are able to induce either full or incomplete maturation of DCs and subsequent induction of Th2 and/or regulatory responses [38,39,40]. In this study, we identified that incubation of ES products from adult *T. spiralis* (AES) and from ML (MES) with mouse BMDCs strongly inducing the expressions of CD40, CD80, CD86, and MHCII on the surface of CD11c^+^ BMDCs, indicating the BMDCs were primed by the *Ts*ES to the status of full maturation. The cytokine profile secreted by the primed DCs showed that AES mainly induced IL-10, TGF-β, and IL-12/23p40. IL-10 and TGF-β are the major regulatory cytokines. IL-12/23p40 plays a critical role in differentiating Th1 and in stabilizing the Th17 phenotype [41]. However, MES-stimulated DCs secreted a variety of Th1/Th2/Treg cytokines such as IL-1β, IL-4, IL-5, IL-6, IL-10, IL-12/23p40, TGF-β, IFN-γ, and TNF-α. The results indicate that both AES and MES not only induce the maturation of DCs but also induce them to secrete high levels of regulatory cytokines IL-10 and TGF-β in addition to other Th1/Th2 cytokines. No significant increase of IL-12 in DCs stimulated using MES antigens was observed by Ilic N. et al. [42]. The possible reason for the different level of IL-12 stimulation compared to Ilic’s results is the different DCs. We used mouse BMDCs while Ilic used human peripheral blood mononuclear cell (PBMC)-derived DCs. AES- and MES-pulsed DCs can create a microenvironment of regulatory cytokines that may stimulate the differentiation of Treg cells. It is consistent with other studies that showed *T. spiralis*-produced antigens induced the full maturation of DCs [43,44]. A recent study reported that GM-CSF-derived BMDCs may contaminate monocyte-derived macrophages in addition to the conventional CD11c^+^MHCII^+^ DCs [45]. We cannot exclude the possible contamination of macrophages in our culture study; however, the highly increased expressions of CD40, CD80, and CD86 on CD11c^+^MHCII^+^ cells, indicating at least the DC portion in the complex, were stimulated by *T. spiralis* ES products. However, further in vivo activation of DCs by *T. spiralis* infection or worm-derived ES products is needed to confirm the conclusion.

To investigate whether *Ts*ES-pulsed DCs have the ability to process and present antigens to help T cells, the sensitized CD4^+^ T cells collected from *T. spiralis*-infected mouse were co-cultured with AES or MES-pulsed BMDCs. Results showed that the *T. spiralis*-infected mouse CD4^+^ T cells were highly proliferated, accompanied by the secretion of cytokines IL-10, TGF-β, IFN-γ, and IL-4, thus confirming that both AES- and MES-activated DCs have the ability of antigen presentation. DCs can modulate immune responses through diversified mechanisms, for example, production of cytokines, suppression of pro-inflammatory mediators, expression of costimulatory molecules, and especially polarization of Tregs [46,47]. Stimuli are responsible for this polarization via DCs. In order to determine whether AES and MES possess the immunomodulatory ability to induce Treg differentiation through DCs so as to stimulate the host T cells regulatory network, the AES or MES was used to pulse DCs and the AES- or MES-pulsed DCs were co-incubated with naive CD4^+^ T cells. As the results with immunization of AES or MES in vivo, the AES- and MES-pulsed DCs were also enabled to induce CD4^+^CD25^+^Foxp3^+^ Treg cells in vitro, accompanied by higher levels of IL-10 and TGF-β, which are mostly secreted by Tregs [48], indicating that AES- or MES-pulsed DCs can modulate host immune responses with differentiation of Tregs, possibly by creating a regulatory cytokine microenvironment or by presenting *T. spiralis* excretory–secreted antigens to CD4^+^ T cells. Interestingly, we observed that the AES upregulated TGF-β expression to a greater extent than MES did, whereas MES had greater influence on enhancing IL-10 than AES did. It is possible that IL-10 and TGF-β are stimulated by different components in the ES products that cause the different levels of both regulatory cytokines when stimulated by AES and MES. However, it seems that TGF-β contributes more stimulation to Treg [32] than IL-10 does in this study.

Although it was confirmed that *T. spiralis* ES antigens possessed the ability to immunomodulate host immune responses by upregulating Treg cells, the ES products from adult worms or ML have different abilities to simulate different Treg cells. Immunization with AES induced both CD4^+^CD25^+^Foxp3^+^ Tregs and CD4^+^CD25^−^Foxp3^+^ Tregs associated with higher level of TGF-β, but MES mainly induced CD4^+^CD25^−^Foxp3^+^ Tregs associated with higher levels of IL-10 and IL-5. It has been confirmed that Foxp3 expression rather than CD25 is essential for Treg suppressive activity [49]. However, CD4^+^CD25^−^ effector T cells have better restriction to inflammation than CD4^+^CD25^+^ T cells during *T. spiralis* chronic infection [33]. The specific stimulation of CD4^+^CD25^−^Foxp3^+^ Treg cells with ES probably suggests that *T. spiralis* ES antigens may be involved not only in the Treg differentiation but also in the control of inflammation during *T. spiralis* chronic infection. The different levels of different types of Tregs and cytokines may result from the different components of ES secreted by the different stages of the nematode. At the adult stage, as a strategy of survival, the parasite secreted more immunomodulatory products that induced a higher level of Tregs to reduce the immune attack to the adult parasite in the intestine. When the larvae migrate to muscle to form larval capsules, it may not be so important to induce the regulatory inhibition to escape from host immune attack because the larvae acquire the protection by the isolation of capsules in muscle.

The *Ts*ES-induced host Treg differentiation possibly through activating DCs was also confirmed by the passive transfer of AES- or MES-pulsed BMDCs to naive mice. The recipient naive mice acquired strong Treg response and Th2 response, indicating that *T. spiralis* ES products stimulate Treg cell differentiation and Th2 polarization possibly through programming DCs. Our results are consistent with other studies for mice passively transferred with *T. spiralis* antigen-activated DCs that induced immunomodulatory effects to reduce inflammatory encephalomyelitis [50]. *T. spiralis* ES products were also used to alleviate DSS-induced colitis by inducing Treg expressing IL-10 and TGF-β.

Additionally, mice immunized with AES also induced significantly increases of Th1 and Th2 cells, consistent with characteristics of *T. spiralis* infection in the intestinal phase. AES caused slight inhibition of Th17 response, probably resulting from the induction of Foxp3^+^ Tregs. Unlike infection of ML phase that did not show Th1 response, MES induced both Th1 and Th2 responses during immunization, possibly because ML was hidden within the capsule structure that reduce MES from exposure to host immune system.

Even though we provided some evidences in this study showing that *T. spiralis*-produced ES products, especially AES, induced differentiation of regulatory T cell response through activating DCs in vitro, there are still a lot that are not clear and need to be further explored, for example, whether the regulatory effects of AES on DCs identified in vitro could be confirmed in vivo; what are the detail receptors on DCs that AES activate and the following pathways that lead to the regulatory property? What are the specific components in the ES products that are involved in the regulatory activation of DCs and other immune cells? These questions are listed on our agenda to be investigated. We believe parasitic helminths secrete some molecules, including proteins, peptidoglycans, or glycolipids that modulate and reduce host immune response, including the activation of regulatory DCs, as a survival strategy. Actually, some proteins have already been discovered to be involved in the regulatory property during *T. spiralis* infection. One of these proteins is *T. spiralis*-secreted paramyosin (*Ts*Pmy) [51]. It has been identified that *Ts*Pmy modulates host immune response not only by binding to host complement component to reduce complement-involved immune attack [20] but also by activating the regulatory property of DC to stimulate Treg response [22]. The recombinant *Ts*Pmy protein was able to induce the differentiation of Treg cells by activating mouse bone marrow-derived DCs, similar to the results we found in AES in this study. However, the interaction between helminth and host, especially the immunomodulatory effects, should be mediated by a complex of molecules secreted by parasitic helminth; *Ts*Pmy may be just one of them involved in this function. More efforts are being paid to identify other molecules playing this role as targets for vaccine development.

## 5. Conclusions

In conclusion, as part of survival strategy, *T. spiralis* infection induces strong host regulatory T cell responses characterized by increasing CD4^+^CD25^+^Foxp3^+^ and CD4^+^CD25^−^Foxp3^+^ Treg cells accompanied by high levels of IL-10 and TGF-β. The similar Treg responses induced by immunization of *T. spiralis* AES and MES indicated that *T. spiralis* infection induces differentiation of Treg through parasite-ES antigens. Both *T. spiralis* AES and MES could activate BMDCs in vitro to facilitate their maturation and to create regulatory cytokines IL-10 and TGF-β. The *T. spiralis* AES- and MES-pulsed DCs possess the abilities not only to present antigens to sensitized CD4^+^ T cells to stimulate their proliferation but also to induce naive CD4^+^ T cell to differentiate to Treg secreting IL-10 and TGF-β. The passive transfer of *T. spiralis* AES- and MES-pulsed BMDCs gives naive mice the ability to differentiate Treg cells. *T. spiralis* AES has a greater ability to induce Treg than MES does, but the latter has the ability to induce CD4^+^CD25^−^Foxp3^+^ Treg cells. The results obtained in this study suggested that *T. spiralis* ES products stimulate the differentiation of host regulatory T cells possibly through activating dendritic cells to create a regulatory environment that benefits the survival of the parasite in the host. However, the *T. spiralis* ES product-induced BMDC differentiation into the regulatory environment observed in in vitro experiments should be further confirmed by DC stimulation in vivo, and the specific components in the *T. spiralis* ES products that play these roles should be further investigated.

## Figures and Tables

**Table 1:** Four pairs of specific primers used for quantitative real-time PCR.

Primer	Forward Sequence	Reverse Sequence
Foxp3	5′-GGCCCTTCTCCAGGACAGA-3′	5′-GCTGATCATGGCTGGGTTGT-3′
TGF-β1	5′-ACCATGCCAACTTCTGTCTG-3′	5′-CGGGTTGTGTTGGTTGTAGA-3′
IL-10	5′-CCCTTTGCTATGGTGTCCTT-3′	5′-TGGTTTCTCTTCCCAAGACC-3′
HPRT 1	5′-AGCCTAAGATGAGCGCAAGT-3′	5′-TTACTAGGCAGATGGCCACA-3′

## Supplementary Materials

The following are available online at https://www.mdpi.com/2073-4409/8/11/1404/s1, Figure S1: Th2, Treg, Th1, and Th17 CD4^+^ T cells from the spleen at different stages post *T. spiralis* infection (intestinal phase, day 6; NBL migration, day 15; larva capsule, day 30; and convalescent phase, day 60) were measured by FACS; Figure S2: Mouse BMDCs surface markers (CD40, CD80, CD86, and MHC II) were detected by FACS after which were pulsed with PBS, BSA, LPS, MES, or AES for 48 h. Figure S3: SDS-PAGE profiles of ES antigen of *T. spiralis.*
