# Supplementary figures and images for: Trichinella spiralis Excretory–Secretory Products Stimulate Host Regulatory T Cell Differentiation through Activating Dendritic Cells
Source: Cells. 2019 Nov 7;8(11):1404. doi: 10.3390/cells8111404 (PMC6912532; doi:10.3390/cells8111404)

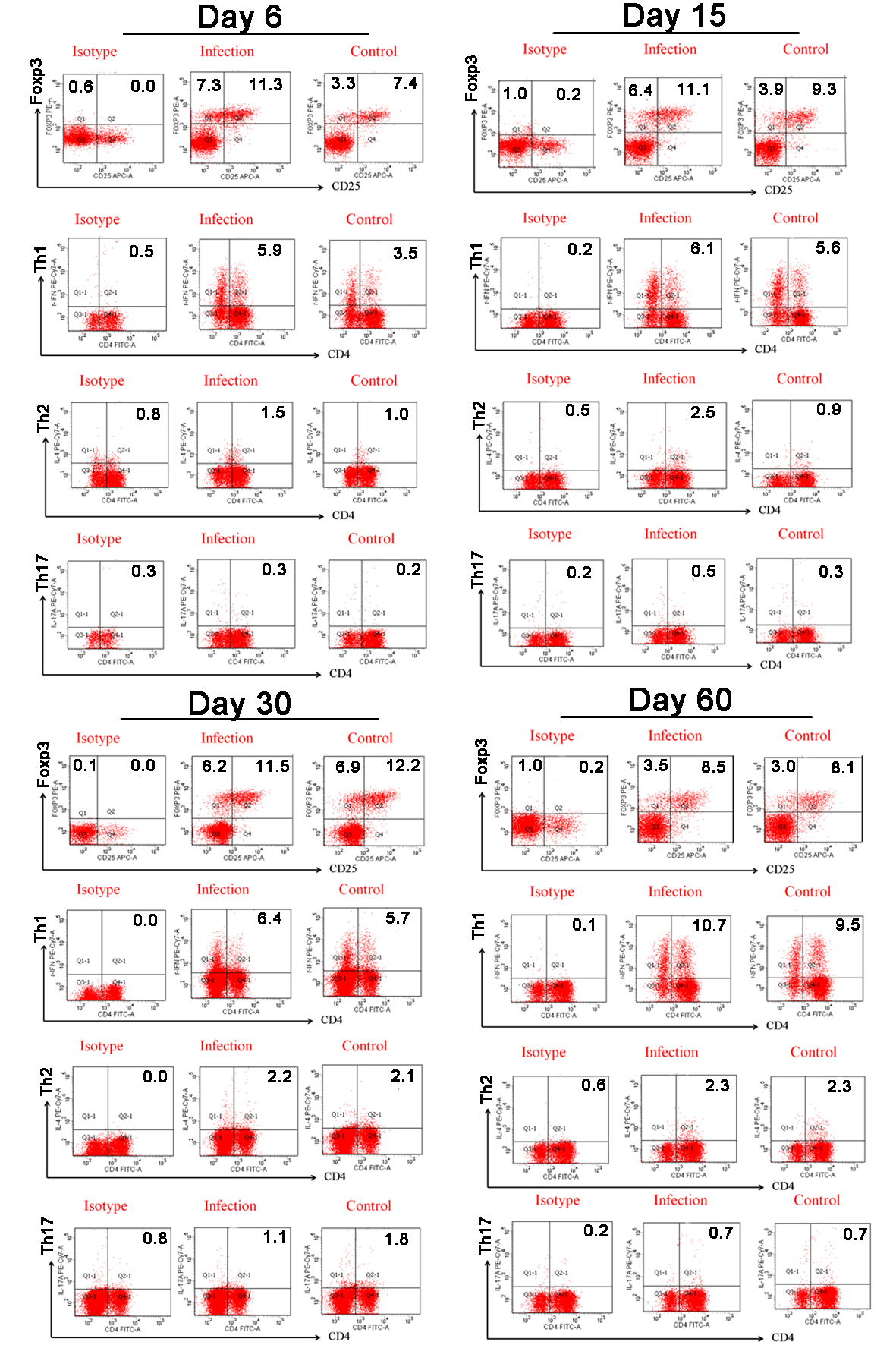

Supplement: Supplementary file 1 [file cells-08-01404-s001.zip › supplementary materials 20190906/FIGURE S1.tif]

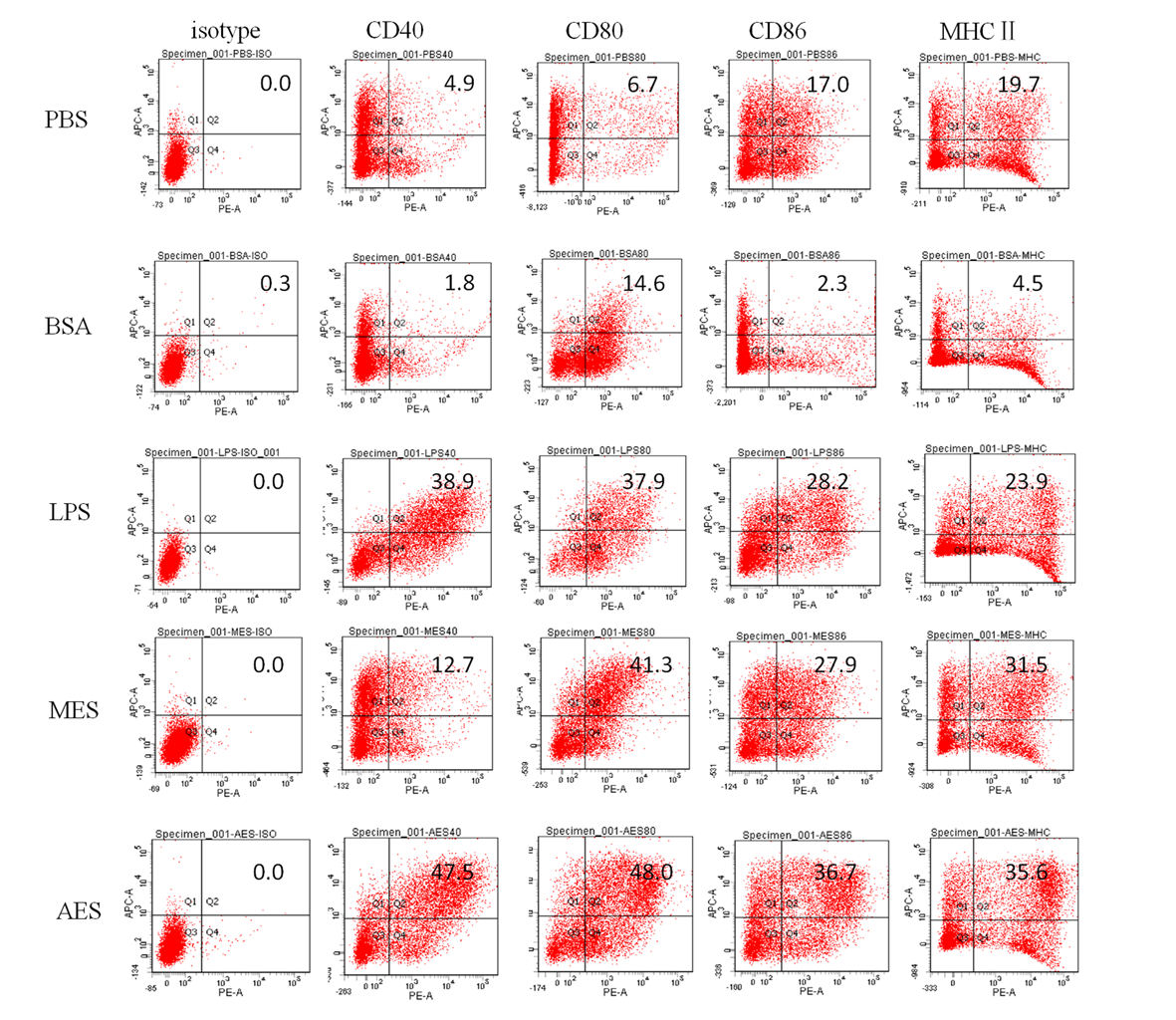

Supplement: Supplementary file 1 [file cells-08-01404-s001.zip › supplementary materials 20190906/FIGURE S2.tif]

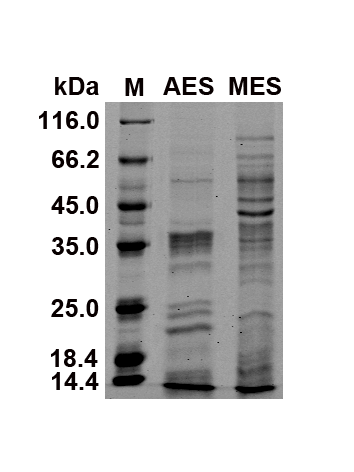

Supplement: Supplementary file 1 [file cells-08-01404-s001.zip › supplementary materials 20190906/Figure S3.tif]
